# MicroRNAs as a Potential Biomarker in the Diagnosis of Cardiovascular Diseases

**DOI:** 10.3390/medicina59071329

**Published:** 2023-07-19

**Authors:** Dagmar Kramna, Petra Riedlova, Vitezslav Jirik

**Affiliations:** 1 Centre for Epidemiological Research, Faculty of Medicine, University of Ostrava, 70103 Ostrava, Czech Republic; petra.riedlova@osu.cz (P.R.); vitezslav.jirik@osu.cz (V.J.); 2 Department of Epidemiology and Public Health, Faculty of Medicine, University of Ostrava, 70103 Ostrava, Czech Republic

**Keywords:** microRNA, CVD, biomarker

## Abstract

Cardiovascular diseases (CVD) are the leading cause of death in most developed countries. MicroRNAs (miRNAs) are highly investigated molecules not only in CVD but also in other diseases. Several studies on miRNAs continue to reveal novel miRNAs that may play a role in CVD, in their pathogenesis in diagnosis or prognosis, but evidence for clinical implementation is still lacking. The aim of this study is to clarify the diagnostic potential of miRNAs in some CVDs.

## 1. Introduction

Several scientific publications have reported, at various levels, possible relationships between changes in serum or plasma microRNA (miRNA) expression and the incidence of cardiovascular diseases (CVD) such as atherosclerosis, coronary heart disease, coronary artery disease, acute coronary syndromes and heart failure. The aim of this review is to help reveal the growing current importance of miRNAs, especially in CVD, and to suggest useful miRNAs that can be identified in blood serum or plasma and have the potential to become diagnostic or prognostic biomarkers. The proposed miRNAs should become the subject of further research.

## 2. Cardiovascular Diseases (CVD)

CVD has been the leading cause of death worldwide for the past 20 years, especially in most developed countries. Despite improvements in prevention, the trend in CVD prevalence is still increasing. The World Health Organization reports that CVD caused 17.9 million deaths in 2019, equivalent to 32% of all global deaths. Ischemic heart disease (IHD) is considered the diagnosis with the highest mortality rate worldwide, accounting for 16% of total deaths. Stroke is the second leading cause of death, accounting for approximately 11% of all deaths. Most CVDs can be prevented by avoiding risky behaviors such as smoking, unhealthy diet, obesity, lack of exercise, and alcohol abuse [1,2].

Early detection of CVDs is essential and is based on risk assessment. Risk assessment includes individual patient characteristics and allows for the development of personalized treatment by treating risk factors, lifestyle modification and psychosocial factors together with social determinants [3]. Modification of risk factors alone can achieve up to 50% reduction in CVD mortality, and another 40% of cardiovascular deaths can be influenced by improved treatment [4]. It is important to understand the pathophysiology of CVD at the molecular level to discover new biomarkers for early and correct prevention, diagnosis and prognosis of these diseases. MiRNAs are currently being highly investigated as promising biomarkers, either alone or in combination with other parameters [5]. Several members of the TGF-Beta superfamily proteins like growth differentiation factors GDF15 and GDF11, emerging cardiokines, mitochondrial DNA copy numbers, and other markers found through proteomics related to oxidative stress are considered as other potential candidates for specific biomarkers whose levels provide information on the course of CVD [6].

## 3. MicroRNA

The human genome is written in DNA, with genes as its basic functional unit. In the process of proteosynthesis, these genes are constantly actively transcribed into RNA, which then serves as a matrix for the formation of the proteins. These are protein-coding genes, which we now know constitute only 1–3% of the genome [7]. The rest of the genome used to be referred as “junk DNA” or redundant DNA [8]. However, advances in molecular biology have revealed that most of the RNA that not serving as a matrix for protein formation is not useless but has a variety of regulatory functions. These RNAs have been named non-coding RNAs and can be classified based on length from long non-coding RNAs to the smallest, called microRNAs [7].

**Figure 1:**
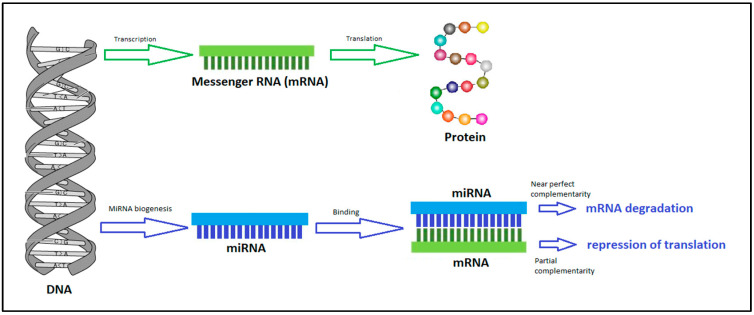
Functions of miRNAs-MiRNAs regulate gene expression by binding to mRNAs leading to mRNA degradation or repression of translation.

MiRNAs are noncoding single-stranded RNA molecules of endogenous origin. Their length corresponds to 20–25 nucleotides. The main function of miRNAs is to regulate gene expression by binding to a target complementary messenger RNA (mRNA) sequence. A single miRNA regulates tens or hundreds of target genes due to a binding region called seed region, which contains only 7–8 nucleotides, and functional consequences can be observed with this binding. The binding between mRNA and miRNA can have a dual consequence (Figure 1) depending on the degree of complementarity of the binding sites, their availability and number. With strong complementarity, mRNA degradation will occur and with imperfect sequence complementarity, translation of the mRNA into the protein will be inhibited. Both cases affect the outcome of gene expression, thus causing a decrease in the level of protein encoded by the target mRNA [9]. In some, but less frequent cases, miRNAs are also able to act in the opposite way, by interacting with the target mRNA to stimulate or activate target proteins [10]. MiRNAs function as gene regulators that exert their function posttranscriptionally, which distinguishes them from classical epigenetic factors (DNA methylation, acetylation, etc.) that regulate gene expression at the chromatin level [11].

MiRNAs represent an extremely fast-growing area of molecular biology, and current research is discovering new roles for miRNAs in the body. Through intensive research, new miRNAs are continuously being discovered every year. There are several databases that collect data of the function or structure of miRNAs. The best-known database is called miRbase. The current version (v22) contains information of miRNAs from 271 organisms, with 2654 entries for mature miRNA sequences and 1917 entries for precursor miRNAs for the human genome [12].

**Figure 2:**
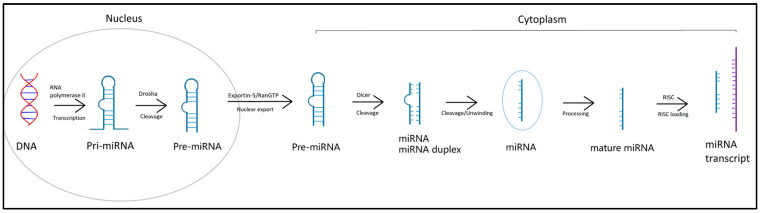
Biogenesis of miRNAs.

The biogenesis of miRNAs occurs in several steps (Figure 2). It starts in the nucleus by transcription of DNA to form primary miRNA transcript (pri-miRNA) using the enzyme RNA polymerase II. Furthermore, subsequent modifications, mainly cleavage, produce precursor-miRNA (pre-miRNA), which is transported into the cytoplasm using the Exportin-5/RanGTP system. The precursor miRNAs have the structure of single-stranded RNA whose ends (3’ and 5’ ends) are linked together. After transport into the cytoplasm, a double-stranded mature miRNA is gradually formed, consisting of a miRNA/miRNA* duplex, where one strand is active, guide and functionally mature and the other strand is inactive sometimes called the passenger strand. Subsequently, one of these strands is degraded, and the other strand complementarily binds to the target mRNA using RNA induced silencing complex (RISC) in the form of a ribonucleoprotein called miRISC or miRNP [5].

It has been found that each miRNA strand can regulate different processes in the same tissue. As an example, miR-21. Up-regulation of the 5p strand (miR-21-5p) is associated with heart failure, while the 3p strand (miR-21-3p) acts paracrine to positively regulate cardiomyocyte hypertrophy [13,14,15]. Recognition of target mRNA occurs by the seed sequence of miRNA, which corresponds to nucleotides in positions 2 to 7 of the chain. Due to the very small size of the seed sequence or due to imperfect base pairing, it is possible that multiple target mRNAs are regulated by only one miRNA and, conversely, one target mRNA may be regulated by multiple miRNA species. Thus, a single miRNA may be involved in the regulation of multiple signaling pathways, independent of each other or functionally linked [16,17].

MiRNAs are located in the intracellular and extracellular space. Intracellular miRNA levels are involved in gene expression and contribute to cell function through their ability to influence the cell cycle, cellular metabolism, and act on signaling within cells. Extracellular miRNAs are found beyond the cell membrane in the bloodstream and most body fluids. In the extracellular space miRNAs are stored in various carriers e.g., exosome, microparticles, lipoproteins, lipid vesicles, etc. [5]. MiRNAs can enter the circulation actively or passively. Actively in the provision of intercellular communication between tissues and passively due to cell necrosis caused by, e.g., ischemia. Their levels thus reflect the state of the organism, tissue damage, or changes in the internal environment caused by disease. These properties argue for the exploration of miRNAs as promising biomarkers for a number of diseases, including CVD [18].

### MicroRNA as a Potential Biomarker

Since the spectrum of potential biomarkers is wide, there are some basic criteria that a new biomarker should meet: 1. high sensitivity and specificity for the disease, 2. availability of the biomarker by a non-invasive method, 3. the ability to detect the disease early, 4. sensitivity to changes during the disease, 5. long half-life in the sample, 6. the possibility of accurate and reliable detection, 7. affordability and another requirement is the clarity for the physician and the patient [18].

MiRNAs meet many of these criteria and are expected to be beneficial for diagnostics. They are characterized by high stability and accurate detection with high sensitivity and specificity due to sequence-specific amplification. Circulating miRNA molecules were first identified in blood, but subsequent studies have revealed their presence in all known body fluids. This guarantees a less invasive test, as most studies show miRNAs in plasma, i.e., only blood sampling would be sufficient for diagnosis. As mentioned above, a portion of the extracellular miRNA is packaged into various carriers after synthesis and enters the bloodstream in this form. Packaging in the carrier ensures high stability of the miRNA even during long-term storage and also resistance to harsh environmental conditions, which can be high temperature or repeated freezing and thawing, extreme pH, or protection from ribonuclease activity, which causes miRNA degradation. Exogenous free miRNAs do not have this protection and are easily degraded in the bloodstream. Most miRNAs are produced by blood cells and tissues such as the heart, liver, lungs, or kidneys [5,19].

The potential use of biomarkers in KVO was discovered several years ago and their clinical incorporation has been the subject of many studies. The diagnostic power of miRNAs is superior or comparable to established biomarkers. Detection of miRNAs in combination with established biomarkers in CVD would improve diagnostic accuracy. At the same time, miRNAs may be useful in the prognosis of future cardiovascular events. In addition, the utility of miRNAs as therapeutic agents for non-cardiovascular as well as cardiovascular medicine is being investigated [20].

However, even miRNA as a biomarker has limitations. These are mainly the cost of testing and also variations in their expression, which may not be related only to the pathology of the disease. Variations in the levels of some miRNAs in the population may be due to ethnic or geographical differences, or age and sex, which must be considered in the results [21]. It is certain that miRNAs are critical regulators of cardiovascular function and play an important role in many aspects of cardiovascular biology, but currently circulating miRNAs are not part of clinical practice. Reasons for this are the contrasting results of some studies, which may be due to differences in research methodologies and failure to include confounding factors, technological requirements, and non-standardized normalization of expression levels. The clinical utility of miRNAs as biomarkers must be supported by mechanistic studies of effect, validation in larger and well-characterized cohorts with normalization of strategies [22].

## 4. Changes at The MicroRNA Level in Cardiovascular Diseases

Published data for the following chapters and for this review in general were identified by searching and selecting from the article lists of Web of Science, Pubmed and Scopus databases. A multi-stage approach was used in the search. First, articles were identified in the search using the keywords “cardiovascular diseases” and “miRNA”. In a second step, the keywords “biomarker”, “circulating”, “coronary heart disease”, “coronary artery disease”, “acute coronary syndromes” and “heart failure” were added. Subsequently, a filter was performed over the time span of the last 15 years.

### 4.1. Atherosclerosis and Coronary Heart Diseases (CHD)

Ischemic heart disease is a group of pathological conditions in which myocardial ischemia or myocardial infarction occurs. Clinically, we distinguish different forms from chronic stable angina to acute coronary syndromes such as unstable angina (AP) or myocardial infarction (MI), and others. Reduced flow through the arteries is most often due to atherosclerosis. Atherosclerosis is a chronic inflammatory disease of the vessel wall characterized by the accumulation of lipids or atherosclerotic plaques [23]. Progressive plaque growth causes stenosis of the arterial lumen, while rupture of an unstable plaque carries the risk of thrombus or complete obstruction of the lumen. All cellular components involved in plaque and thrombus formation (platelets, monocytes, and endothelial cells in all stages) can release miRNAs, and at the same time, several miRNAs influence the different pathophysiological processes of atherosclerosis (lipoprotein and cholesterol metabolism, inflammatory response, and vascular remodeling). This suggests that miRNAs could serve as a useful biomarker of IHD, both acute and chronic forms [24].

Currently, the diagnosis of coronary artery disease is based on the evaluation of the medical history and includes physical and other examinations (electrocardiogram, echocardiogram, scintigraphy, coronary angiography, or evaluation of the cardiac enzyme). These examinations can be used to determine the myocardial response to stress and to demonstrate ischemia. Coronary angiography remains the gold standard for diagnosis and therapy and is the most invasive of these methods [25]. The diagnosis may also include an overall risk assessment using a risk assessment system [26]. The SCORE system (Systematic Coronary Risk Evaluation system) is one of the most widely used systems in the Czech Republic, but also in Europe, and allows estimation of the risk of CVD death over the next 10 years using 5 factors: sex, age, smoking, total cholesterol, and systolic blood pressure [27,28].

Uncovering some of the relationships between miRNAs and the pathophysiological processes of CVD allows for an easier search for potential biomarkers. There are already known miRNAs that regulate cholesterol transport and lipoprotein metabolism (Table 1), including miR-33, miR-27b, miR-148a or miR-223 [29]. These miRNAs are involved in cholesterol uptake by HDL particles (miR-33, ABCA1 protein) [29], cholesterol transport through hepatocytes into the bile ducts (miR-33, ABCB11 transporter) [30], in the formation of lipoproteins (miR-27, e.g., apolipoprotein B required for the formation of VLDL and LDL) [31] or in the uptake of LDL lipoproteins also by the scavenger receptor (miR-148a [32] and miR-223 [33]). Experimental studies have obtained interesting results that blocking miR-148a increased the clearance of LDL particles in liver tissue [32] and blockade of miR-33 increased HDL levels by up to 50% while reducing atherosclerotic plaques [34]. The course of atherosclerosis is also influenced by miR-126-5p, through its ability to regulate endothelial cell function and increase endothelium regeneration itself. Studies in patients with IHD indicate significantly lower plasma levels of miR-126-5p in association with the formation of atherosclerotic plaques [35].

One of the first studies to examine circulating miRNAs in patients diagnosed with coronary artery disease is a 2010 cohort study led by Fichtischerer. The study results showed that the expression of miR-17, miR-126, miR-92a, mir-145 and miR-155 were significantly down-regulated in patients with coronary heart disease. The opposite trend of increased expression was demonstrated for miR-133 and miR-208a [26].

Another cohort study by Wang et al. presents miR-31, miR-720a, and vasohibin 1 as potential diagnostic biomarkers for the early stages of coronary disease. The results showed lower levels of these miRNAs in patients with CHD compared to patients without the disease [36]. The opposite trend was also observed for miR-206 and miR-574-5p, whose plasma expressions were significantly up-regulated in patients with coronary artery disease compared to healthy controls [37].

Other miRNAs that could aid in the diagnosis of IHD include circulating miR-765, miR-424, and miR-149, whose plasma levels can be used to distinguish patients with stable and unstable angina from those without the disease [38]. Plasma levels of miR-34a, miR-21, and miR-23a [39] as well as miR-196-5p, miR-3163-3p, miR-145-3p and miR-190a-5p [40] have shown the same ability. Plasma levels of miR-133a have also been identified as a potent biomarker of coronary artery disease, particularly acute myocardial infarction, and the level of this miRNA may reflect the severity of coronary atherosclerosis in patients with IHD [41].

A follow-up step to improve the sensitivity and specificity of the assay was to develop a combined panel of circulating miRNAs to better identify patients with IHD compared to evaluating the levels of specific miRNAs alone. This study was carried out by Faccini and collective, where three miRNAs, namely miR-155, miR-145 and a precursor miRNA called let-7c, were combined based on previous studies. The combination of these three miRNAs confirmed the potential for the diagnosis of coronary disease [42]. The aforementioned miRNAs together with their diagnostic potential and changes in level of expression are shown in Table 2.

### 4.2. Acute Coronary Syndrome—Myocardial Infarction (MI) and Unstable Angina Pectoris (AP)

One of the leading causes of CVD death is acute myocardial infarction (AMI). AMI is defined as a state of myocardial injury due to myocardial necrosis in a clinical setting corresponding to myocardial ischemia [43]. After undergoing AMI, cardiomyocyte death occurs, leading to the release of miRNAs into the circulation.

The current diagnosis of acute forms of IHD is based on symptoms and signs, electrocardiogram and cardiac markers. These examinations allow to differentiate between unstable angina and myocardial infarction with or without elevation of the ST segment. This is essential information for the deployment of the correct treatment. The cardiac markers most commonly monitored are troponins I or T (cTnI or cTnT) and myoglobin, which are released into the bloodstream when the myocardium is damaged. The diagnosis can be supplemented by determining the levels of the cardiac creatine kinase isoenzyme (CK-MB) [44]. The problem is that these biomarkers do not increase consistently within the first few hours after symptom onset, and therefore measurements need to be repeated subsequently. Troponins are released into the bloodstream very early, 3 to 9 h after the infarction. Troponin cTnI has an increased level in 4 to 6 h with a peak in 12 h and normalizes in 3 to 10 days, whereas troponin cTnT increases for 12 to 48 h and returns to normal values in 10 days. CK-MB increases in the bloodstream 4 to 6 h after the onset of chest pain and reaches peak levels between 10 and 12 h after MI. Myoglobin is released most rapidly within 1 h and peaks around 8 to 10 h, returning to normal levels within 24 h. This situation of different time release makes immediate diagnosis impossible [45,46].

The most abundantly expressed miRNAs in cardiac tissue include miR-1, miR-133a, miR-208, and miR-499. These miRNAs are important for the development and proper functioning of the myocardium, and therefore their dysregulation is associated with the occurrence and progression of heart disease. Specifically, miR-1 together with miR-133a control the early stages of cardiogenesis and control cardiac electrical conduction in the adult, and miR-208 with miR-499 control the late cardiogenic stages and together regulate the expression of sarcomeric contractile proteins. This is a group of miRNAs that is increasingly being proposed in studies as a biomarker of heart disease [47]. This group of miRNAs is released into the bloodstream in patients with AMI due to cardiomyocyte damage, among other factors, and therefore up-regulation of their plasma levels can be observed [48].

MiRNAs that are highly expressed in the heart can be used to diagnose coronary syndrome, ideally in combination with traditional CVD biomarkers. A 2015 systematic review selected three of 52 different miRNAs that have been studied most extensively and confirmed to have diagnostic or prognostic statistical significance for different CVD at different stages of the disease. These were miR-133a/b, miR-208a/b, or miR-499. The search also mentioned a potential biomarker for acute coronary syndrome, namely miR-1 and miR-145b, with miR-1 having a higher sensitivity for all types of AMI and miR-145 for STEMI and worse AMI courses [49]. For example, other studies have reported miR-155-5p, miR-483-5p, and miR-451, which can be used to distinguish plaque rupture with significant classification power [50].

Studies often compare miRNAs with diagnostic biomarkers that are considered traditional for the diagnosis of CVD. Examples include mir-19a, whose circulating levels indicate a much more reliable diagnosis compared to traditional CVD biomarkers such as creatine kinase (CK) and creatine kinase-MB (CK-MB), myoglobin (MYO), high-sensitivity cardiac troponin I (hs-TnI) and brain natriuretic peptide (BNP) [51]. A combined panel of 3 miRNAs including miR-132, miR-150 and miR-186 achieved high diagnostic precision for unstable AP, whereas the classical biomarker troponin I or even a combination of four classical biomarkers (troponin I, BNP, C-reactive protein and cystatin C) did not have such diagnostic values. An approach could provide more clinically useful information compared to the evaluation of single miRNAs alone [52].

The dynamics of these cardiac-specific miRNAs have also been investigated and it has been found that there is an increase in plasma levels with a peak within 12 h of MI. Peak levels of miR-208b were correlated with troponin T levels and overall cardiac function, demonstrating a potential role for these molecules as a potential tool in the diagnosis of acute coronary syndrome, as well as in predicting the risk of long-term complications [53].

MiR-1, miR-21, and miR-499 have been shown to increase the diagnostic value of the highly sensitive cardiac damage marker troponin (hs-Tn) or even their combination has been evaluated as a statistically better biomarker for diagnosis than hs-Tn. In this study, the dynamics of miRNA levels were also observed in patients with acute IHD. MiRNA expressions already increased in the early stages when troponin was negative. Troponin levels have been reported to remain negative in patients with unstable AP and increase with increasing time in patients with MI, therefore they may still be negative too soon after symptoms. Since miRNA levels were elevated earlier than troponin, it could therefore have the potential to improve the prognosis of patients by early initiation of treatment [54]. 

Studies that aimed to identify a marker for early and accurate diagnosis of AMI or unstable AP identified miR-208a/b as a suitable biomarker [48,55,56] even when compared to cTnI or hs-cTnT, as well as miR-1, miR-133a [48,56], miR-320a [55] and miR-499 [48,55,57,58]. Furthermore, a difference was observed between the expression of miRNAs (miR-1, miR-133, miR-208b) in patients with AMI and unstable AP. Patients with AMI showing higher levels of these miRNAs [56].

Different miRNA expressions can be observed in different groups of AMI, i.e., STEMI or non-STEMI. Higher levels have been described in STEMI patients for miR-133a, miR-208b, miR-451, miR-499 and miR-134 compared to non-STEMI cases [55,59]. The exception is miR-145, which showed the opposite trend and was lower in STEMI than in other patient groups [60].

The miRNA also has the potential to be used to predict the future development of the patient after AMI. A study focused on prognostic impact evaluated serum levels of 667 miRNAs in patients who experienced AMI. The most significant findings were miR-155 and miR-380, whose serum levels were 4- and 3-fold higher in patients who experienced cardiac death within 1 year of discharge compared to patients who did not experience any cardiovascular events during the subsequent 3 years after discharge. Therefore, patients at risk for cardiac death can be identified [61]. The predictive value of contractility impairment in patients after AMI was increased by a panel of 4 miRNAs, namely miR-16, miR-27a, miR-101 and miR-150, which were evaluated together with the N-terminal promoter natriuretic peptide (Nt-proBNP) [62]. To evaluate prediction and prognosis, the HUNT study reported a set of five miRNAs, namely miR-106a-5p, miR-424-5p, let-7g-5p, miR-144-3p and miR-660-5p in relation to the detection of future IM [63]. Another large-scale study in a cohort of 1112 patients called AtheroGene identifies 7 circulating miRNAs that predict mortality and highlights in particular miR-132, miR-140-3p and miR-210 which were significant predictors of cardiovascular death [64]. Serum miR-126, miRNA-197 and miRNA-223 were also investigated for predicting future MI in a cohort of 820 individuals from the Bruneck study [65]. The results of reliable prediction were validated by a recent study that confirmed the importance of miR-197 and miR-223 as predictors of cardiovascular death in a large cohort of CAD patients [66]. The last four studies were clinical miRNA studies with high numbers of probands and validated data. MiRNAs and their role in acute coronary syndrome are mentioned in Table 3.

### 4.3. Heart Failure (HF)

Heart failure is a clinical syndrome resulting from loss of compensatory pumping function of the heart due to functional and/or structural abnormalities of the heart, with typical symptoms including dyspnea, swelling, malaise and reduced performance [67]. There are several causes and risk factors for the disease, including coronary artery diseas, hypertension, diabetes mellitus, myocardial infarction, atrial fibrillation and others. A common cause of heart failure is coronary heart disease or cardiomyopathy. The prevalence of heart failure in developed countries is reported to be 1 to 2% of the population, while the prevalence can be as high as 10% in those over 70 years of age. Imaging methods are used for diagnosis, which can be supplemented by examination of the natriuretic peptides BNP and NT-proBNP [68].

The publication summarizing the findings of 21 cohort studies investigating the importance of miRNAs in HF highlighted selected miRNAs out of 71 that showed changes in expression in at least two independent studies. These were miR-1, miR-21, miR-30a, miR-92a, miR-126, miR-150, miR-195, miR-210, miR-342-3p, miR-423-5p, miR-499-5p and miR-622. Evaluation of dysregulation of these miRNAs in the context of HF may help identify key mechanisms of pathogenesis of HF [69]. Moreover, some of these selected miRNAs seem to regulate important genes involved in cardiac remodeling. For example, mir-1 and miR-30a target genes playing a role in cardiac apoptosis and hypertrophy [70,71,72,73] miR-92a, miR-195 and miR-499-5p target genes involved in apoptosis signaling [74,75,76] and miR-21 targets molecules in signaling pathways controlling cardiac apoptosis, hypertrophy and fibrosis [14,71,77].

Some of these and other miRNAs have subsequently been mentioned in relation to HF in other studies. The same result was obtained for plasma levels of other miRNAs-miR-18b, miR-1254, miR-129-5p, miR-622, miR-675 and miR-423-5p [78] miR-22, miR-92b, miR-320a [79]. The strongest correlation for the diagnosis of heart failure was found for miR-423-5p [78,79], but Tutarel et al. did not confirm this correlation in subjects with right-sided heart failure [80]. Circulating miR-150-5p was also identified as a biomarker for advanced heart failure [81].

The performance of the miRNA panel for HF detection and categorization of HF subtypes was evaluated. Three detection options were compared: 1. using NT-proBNP, 2. using a panel of 8 miRNAs, and 3. a combination of these parameters. The third option and thus the combination of NT-proBNP with panel 8 miRNAs proved to be the most diagnostically powerful with improved specificity and accuracy. The number of miRNAs was also investigated during the introduction of the panel. The panels contained from 3 to 8 miRNAs and a number of 8 miRNAs was selected as optimal for HF detection [82].

As in other diagnoses, miRNAs could have a high potential in the prognosis of heart failure. Studies have shown the strong prognostic properties of miRNA-21 and miR-132, the levels of which predict cardiovascular mortality and the risk of patient rehospitalization [83,84]. The aforementioned miR-423-5p has been identified as a prognostic marker and its level correlates with readmission to the hospital and higher mortality. Such changes are indicative of long-term poor outcomes in patients with heart failure [85]. A prospective cohort study of patients diagnosed with chronic heart failure repeatedly monitored miR-22-3p levels and found a correlation between miRNA levels and adverse outcomes, including hospitalization, CVD mortality, heart transplantation, or implantation of left ventricular assist devices. Based on this study, miR-22-3p may be considered an important predictor of prognosis in people with chronic heart failure [86].

Another important marker predicting cardiovascular mortality was mir-182, and interestingly, its predictive power was higher than that of sensitive C-reactive protein and NT-proBNP [87].

Among other things, miRNAs could find their essential application in patients in the terminal stage of HF who have had to undergo heart transplantation, where there is a subsequent risk of acute cellular rejection (graft rejection). Serum levels of circulating miRNAs, specifically miR-10a, miR-31, miR-92a and miR-155, showed differential serum expression that was consistent with tissue expression. These four miRNAs highly discriminated patients with rejection from those without [88]. As the risk of rejection is currently assessed at several routine visits by invasive endomyocardial biopsy, noninvasive miRNA monitoring could be of considerable benefit in the future [88,89].

It is important to mention that some studies have not yet considered miRNAs to be a sufficient diagnostic or pathophysiological biomarker of HF. An example is a 2020 systematic review that concluded that miRNAs currently lack sufficient support for use in the clinical setting and further research is needed on this issue [90]. The role of individual miRNAs in heart failure is shown in Table 4.

## 5. Conclusions

The field of miRNAs as a potential biomarker is still relatively young, yet it is expected to contribute significantly to the diagnosis and prognosis of diseases, including CVD. MiRNAs bring several advantages to diagnostics due to their low stability, high sensitivity and specificity as well as lower invasiveness of sample collection. However, there are still disadvantages that may result from incomplete scientific understanding of the role of miRNAs, lack of standardization of expression values across samples with respect to natural co-founding factors in the population. This review provides information on a total of 50 miRNAs detected in plasma or serum that studies have shown to play an important role in CVD and should be the subject of further studies. Since the number of miRNAs is high and their importance is still being uncovered, it is believed that better diagnostic value can be obtained by testing a larger number of miRNAs that will be supplemented with other standard biomarkers, thereby increasing the amount of information about disease diagnosis or prognosis.

## Figures and Tables

**Table 1:** Pathophysiological significance of miRNAs in atherosclerosis and IHD.

MiRNA	Diseases	Pathophysiological Significance	Ref.
miR-33	Atherosclerosis	Uptake of cholesterol by HDL particles Transport of cholesterol through hepatocytes into the bile ducts	[29,30,34]
miR-27b	Atherosclerosis	Formation of lipoproteins	[31]
miR-148	Atherosclerosis	LDL lipoprotein uptake	[32]
miR-223	Atherosclerosis	LDL lipoprotein uptake by the scavenger receptor	[33]
miR-126, miR-126-5p	IHD, atherosclerosis	Regulation of endothelial cell function and enhancement of endothelial regeneration capacity Lower plasma miR-126-5p levels are associated with atherosclerotic plaque formation	[35]

miRNAs-microRNAs, IHD—ischemic heart disease, HDL—high density lipoprotein, LDL—low density lipoprotein.

**Table 2:** Diagnostic potential of miRNAs for IHD.

MiRNA	Diagnostic Potential	MiRNA Alteration	Ref.
miR-17	IHD biomarker	↓	[26]
miR-92a	IHD biomarker	↓	[26]
miR-126	IHD biomarker	↓	[26]
miR-145	IHD biomarker	↓	[26]
miR-155	IHD biomarker	↓	[26]
miR-133, miR-208a	IHD biomarker	↑	[26]
panel miR-155, miR-145 and let-7c	IHD biomarker	↓	[26]
miR-31, miR-720a	Biomarker of the early phase of IHD	↓	[36]
miR-206, miR-574-5p	Biomarker of the early phase of IHD	↑	[37]
miR-149 miR-765, miR-424	IHD biomarker-differentiating patients with stable and unstable AP from those without the disease	↑ ↓	[38]
miR-21, miR-23a, miR-34a	Biomarker of IHD and disease progression	↑	[39]
miR-145-3p, miR-190a-5p miR-196-5p, miR-3163-3p	Biomarker of the early phase of IHD	↓	[40]
miR-133a	Prediction of the presence and severity of coronary lesions in patients with IHD Biomarker AMI	↑	[41]

miRNA—microRNA, IHD—ischemic heart disease, AMI—acute myocardial infarction, ↓—down-regulation, ↑—up-regulation.

**Table 3:** Diagnostic potential of miRNAs for MI and AP.

MiRNA	Diagnostic Potential	MiRNA Alteration	Ref.
miR-499	Diagnostic or prognostic significance for all CVD Biomarker AMI Distinguishing STEMI and non-STEMI AMI	↑ ↑ STEMI	[47,48,49,54,55,58,59]
miR-208a/b	Diagnostic or prognostic significance for all CVD Biomarker AMI Differentiation between STEMI and non-STEMI AMI Biomarker AP and differentiation between AMI and AP Prognostic marker of long-term complications after AMI	↑ ↑ STEMI	[47,48,49,54,55,56,59]
miR-1	Biomarker AMI Biomarker AP-differentiation of AMI and AP	↑	[47,48,49,54,55,56]
miR-133a/b	Diagnostic or prognostic significance for all CVD Biomarker of AMI and AP Distinguishing AMI and AP Distinguishing STEMI and non-STEMI AMI	↑ ↑ STEMI	[47,48,49,55,56]
miR-145	Biomarker of STEMI and worse AMI Distinguishing STEMI and non-STEMI AMI	↓ ↓ STEMI	[49,60]
miR-451	Recognition of plaque rupture Recognition of STEMI and non-STEMI AMI	↓ ↑ STEMI	[50,55]
miR-155, miR-155-5p	Prognostic impact-risk of cardiac death after AMI Recognition of plaque rupture	↑	[50,61]
miR-483-5p	Recognition of plaque rupture	↑	[50]
miR-19a	Biomarker AMI	↑	[51]
Panel: miR-132, miR-150, miR-186	Early diagnosis of unstable angina	↑	[52]
miR-21	Biomarker AMI	↑	[54]
miR-320a	Biomarker AMI	↑	[55]
miR-134	Distinguishing STEMI and non-STEMI AMI	↑ STEMI	[59]
miR-380	Prognostic impact-risk of cardiac death after AMI	↑ in patients at risk of cardiac death	[61]
Panel: miR-16, miR-27a miR-101, miR-150	Prognostic impact-risk of contractility failure after AMI	↑ ↓	[62]
miR-106a-5p, miR-424-5p, let-7g-5p, miR-144-3p, miR-660-5p	Prediction of future IM	↑	[63]
miR-132, miR-140-3p, miR-210	Predictors of cardiovascular death	↑	[64]
miR-126, miRNA-197, miRNA-223	Prediction of future MI	↑	[65,66]

miRNA—microRNA, AMI—acute myocardial infarction, AP—angina pectoris, CVD—cardiovascular disease, STEMI—ST Elevation Myocardial Infarction, IM—myocardial infarction, ↓—down-regulation, ↑—up-regulation.

**Table 4:** Diagnostic potential of miRNAs for heart failure.

miRNA	Diagnostic Potential	MiRNA Alteration	Ref.
miR-423-5p	Diagnosis of HF Prognostic biomarker of acute HF	↑ ↓	[69,78,79,85]
miR-622	Biomarker HF	↑	[69,78]
miR-92a/b	Diagnosis of HF and correlation with important clinical prognostic parameters Non-invasive biomarker for risk of heart transplant rejection	↑	[69,79,88]
miR-150, miR-150-5p	Biomarker of advanced HF, association with maladaptive remodelling, disease severity	↓	[69,81]
miR-21	HF biomarker and prediction of cardiovascular mortality and risk of rehospitalization	↑	[69,83]
miR-1	Biomarker HF	↑	[69]
miR-30a	Biomarker HF	↑	[69]
miR-126	Biomarker HF	↑↓ *	[69]
miR-195	Biomarker HF	↓	[69]
miR-210	Biomarker HF	↑	[69]
miR-342-3p	Biomarker HF	↓	[69]
miR-499-5p	Acute heart failure	↑	[69]
miR-18b	Biomarker HF	↑	[78]
miR-129-5p	Biomarker HF	↑	[78]
miR-675	Biomarker HF	↑	[78]
miR-1254	Biomarker HF	↑	[78]
miR-22, miR-22-3p	Diagnosis of HF and correlation with important clinical prognostic parameters Prediction of prognosis in chronic HF	↑	[79,86]
miR-320a	Diagnosis of HF and correlation with important clinical prognostic parameters	↑	[79]
miR-132	Biomarker HF Predication readmission and risk of rehospitalization	↑ ↓	[84]
miR-182	Prediction of cardiovascular mortality	↑	[87]
miR-10a	A non-invasive biomarker for the risk of heart transplant rejection	↓	[88]
miR-31	A non-invasive biomarker for the risk of heart transplant rejection	↑	[88]
miR-155	A non-invasive biomarker for the risk of heart transplant rejection	↑	[88]

miRNA—microRNA, HF—heart failure, ↓—down-regulation, ↑—up-regulation, * Different expression depending on the biologic material.

## Data Availability

Not applicable.

## References

[1] World Health Organization (2023). “Cardiovascular Diseases”. https://www.who.int/health-topics/cardiovascular-diseases#tab=tab_1.

[2] World Health Organization (2020). “The Top 10 Causes of Death”. https://www.who.int/news-room/fact-sheets/detail/the-top-10-causes-of-death.

[3] Visseren F., Mach F., Smulders Y., Carballo D., Koskinas K., Bäck M., Benetos A., Biffi A., Boavida J., Capodanno D. (2021). 2021 ESC Guidelines on cardiovascular disease prevention in clinical practice. Eur. Heart J..

[4] Cífková R., Vaverková H., Filipovský J., Aschermann M. (2014). Summary of the European Guidelines on cardiovascular disease prevention in clinical practice (version 2012): Prepared by the Czech Society of Cardiology: Prepared by the Czech Society of Cardiology. Cor et Vasa.

[5] Çakmak H., Demir M. (2020). Microrna and cardiovascular diseases. Balk. Med. J..

[6] Rochette L. (2022). Emerging New Biomarkers for Cardiovascular Disease. Int. J. Mol. Sci..

[7] Djebali S., Davis C., Merkel A., Dobin A., Lassmann T., Mortazavi A., Tanzer A., Lagarde J., Lin W., Schlesinger F. (2012). Landscape of transcription in human cells. Nature.

[8] Grant B. (1981). The safe-neighborhood hypothesis of junk DNA. J. Theor. Biol..

[9] Bartel D. (2004). MicroRNAs: Genomics, Biogenesis, Mechanism, and Function: Genomics, Biogenesis, Mechanism, and Function. Cell.

[10] Lytle J., Yario T., Steitz J. (2007). Target mRNAs are repressed as efficiently by microRNA-binding sites in the 5′ UTR as in the 3′ UTR. Proc. Natl. Acad. Sci. USA.

[11] Ambros V. (2004). The functions of animal microRNAs. Nature.

[12] Kozomara A., Birgaoanu M., Griffiths-Jones S. (2019). miRBase: From microRNA sequences to function: From microRNA sequences to function. Nucleic Acids Res..

[13] Treiber T., Treiber N., Meister G. (2019). Regulation of microRNA biogenesis and its crosstalk with other cellular pathways. Nat. Rev. Mol. Cell Biol..

[14] Thum T., Gross C., Fiedler J., Fischer T., Kissler S., Bussen M., Galuppo P., Just S., Rottbauer W., Frantz S. (2008). MicroRNA-21 contributes to myocardial disease by stimulating MAP kinase signalling in fibroblasts. Nature.

[15] Bang C., Batkai S., Dangwal S., Gupta S., Foinquinos A., Holzmann A., Just A., Remke J., Zimmer K., Zeug A. (2014). Cardiac fibroblast–derived microRNA passenger strand-enriched exosomes mediate cardiomyocyte hypertrophy. J. Clin. Investig..

[16] Colpaert R., Calore M. (2021). Epigenetics and microRNAs in cardiovascular diseases. Genomics.

[17] Bartel D. (2009). MicroRNAs: Target Recognition and Regulatory Functions: Target Recognition and Regulatory Functions. Cell.

[18] Siasos G., Bletsa E., Stampouloglou P., Oikonomou E., Tsigkou V., Paschou S., Vlasis K., Marinos G., Vavuranakis M., Stefanadis C. (2020). MicroRNAs in cardiovascular disease. Hell. J. Cardiol..

[19] Szelenberger R., Kacprzak M., Saluk-Bijak J., Zielinska M., Bijak M. (2019). Plasma MicroRNA as a novel diagnostic. Clin. Chim. Acta.

[20] Schulte C., Karakas M., Zeller T. (2017). microRNAs in cardiovascular disease—Clinical application. Clin. Chem. Lab. Med..

[21] Fazmin I., Achercouk Z., Edling C., Said A., Jeevaratnam K. (2020). Circulating microRNA as a Biomarker for Coronary Artery Disease. Biomolecules.

[22] Cavarretta E., Frati G. (2016). MicroRNAs in Coronary Heart Disease: Ready to Enter the Clinical Arena?. BioMed Res. Int..

[23] Jensen R., Hjortbak M., Bøtker H. (2020). Ischemic Heart Disease: An Update. Semin. Nucl. Med..

[24] Churov A., Summerhill V., Grechko A., Orekhova V., Orekhov A. (2019). MicroRNAs as Potential Biomarkers in Atherosclerosis. Int. J. Mol. Sci..

[25] Cohen M.D.B. (2019). Coronary Heart Disease: From Diagnosis to Treatment.

[26] Fichtlscherer S., Rosa S., Fox H., Schwietz T., Fischer A., Liebetrau C., Weber M., Hamm C., Röxe T., Müller-Ardogan M. (2010). Circulating microRNAs in patients with coronary artery disease. Circ. Res..

[27] Conroy R., Pyörälä K., Fitzgerald A., Sans S., Menotti A., Backer G., Bacquer D., Ducimetière P., Jousilahti P., Keil U. (2003). Estimation of ten-year risk of fatal cardiovascular disease in Europe: The SCORE project: The SCORE project. Eur. Heart J..

[28] Cifkova R., Byma S., Ceska R., Horky K., Karen I., Kunesova M., Kralikova E., Rosolova H., Roztocil K., Soska V. (2005). Prevence kardiovaskularnich onemocneni v dospelem veku. Spolecne doporuceni ceskych odbornych spolecnosti. Vnitr. Lek..

[29] Najafi-Shoushtari S., Kristo F., Li Y., Shioda T., Cohen D., Gerszten R., Näär A. (2010). MicroRNA-33 and the SREBP Host Genes Cooperate to Control Cholesterol Homeostasis. Science.

[30] Allen R., Marquart T., Albert C., Suchy F., Wang D., Ananthanarayanan M., Ford D., Baldán Á. (2012). miR-33 controls the expression of biliary transporters, and mediates statin- and diet-induced hepatotoxicity. EMBO Mol. Med..

[31] Vickers K., Shoucri B., Levin M., Wu H., Pearson D., Osei-Hwedieh D., Collins F., Remaley A., Sethupathy P. (2013). MicroRNA-27b is a regulatory hub in lipid metabolism and is altered in dyslipidemia. Hepatology.

[32] Goedeke L., Rotllan N., Canfrán-Duque A., Aranda J., Ramírez C., Araldi E., Lin C., Anderson N., Wagschal A., de Cabo R. (2015). *MicroRNA*-148a regulates LDL receptor and ABCA1 expression to control circulating lipoprotein levels. Nat. Med..

[33] Vickers K., Landstreet S., Levin M., Shoucri B., Toth C., Taylor R., Palmisano B., Tabet F., Cui H., Rye K. (2014). *MicroRNA*-223 coordinates cholesterol homeostasis. Proc. Natl. Acad. Sci. USA.

[34] Rayner K., Sheedy F., Esau C., Hussain F., Temel R., Parathath S., van Gils J., Rayner A., Chang A., Suarez Y. (2011). Antagonism of miR-33 in mice promotes reverse cholesterol transport and regression of atherosclerosis. J. Clin. Investig..

[35] Li H., Zhao X., Liu Y., Meng Z., Wang D., Yang F., Shi Q. (2016). Plasma MicroRNA-126-5p is Associated with the Complexity and Severity of Coronary Artery Disease in Patients with Stable Angina Pectoris. Cell. Physiol. Biochem..

[36] Wang H., Huang T., Lo H., Huang P., Lin C., Chang S., Liao K., Tsai C., Chan C., Tsai C. (2014). Deficiency of the MicroRNA-31-MicroRNA-720 pathway in the plasma and endothelial progenitor cells from patients with coronary artery disease. Arterioscler. Thromb. Vasc. Biol..

[37] Zhou J., Shao G., Chen X., Yang X., Huang X., Peng P., Ba Y., Zhang L., Jehangir T., Bu S. (2016). miRNA 206 and miRNA 574-5p are highly expression in coronary artery disease. Biosci. Rep..

[38] Sayed A., Xia K., Li F., Deng X., Salma U., Li T., Deng H., Yang D., Haoyang Z., Yang T. (2015). The diagnostic value of circulating microRNAs for middle-aged (40–60-year-old) coronary artery disease patients. Clinics.

[39] Han H., Qu G., Han C., Wang Y., Sun T., Li F., Wang J., Luo S. (2015). MiR-34a, miR-21 and miR-23a as potential biomarkers for coronary artery disease: A pilot microarray study and confirmation in a 32 patient cohort: A pilot microarray study and confirmation in a 32 patient cohort. Exp. Mol. Med..

[40] Du Y., Yang S.H., Li S., Cui C.J., Zhang Y., Zhu C.G., Guo Y.L., Wu N.Q., Gao Y., Sun J. (2016). Circulating MicroRNAs as Novel Diagnostic Biomarkers for Very Early-onset (≤40 years) Coronary Artery Disease. Biomed. Environ. Sci..

[41] Wang F., Long G., Zhao C., Li H., Chaugai S., Wang Y., Chen C., Wang D. (2013). Plasma microRNA-133a is a new marker for both acute myocardial infarction and underlying coronary artery stenosis. J. Transl. Med..

[42] Faccini J., Ruidavets J., Cordelier P., Martins F., Maoret J., Bongard V., Ferrières J., Roncalli J., Elbaz M., Vindis C. (2017). Circulating MIR-155, MIR-145 and let-7c as diagnostic biomarkers of the coronary artery disease. Sci. Rep..

[43] Ibanez B., James S., Agewall S., Antunes M., Bucciarelli-Ducci C., Bueno H., Caforio A., Crea F., Goudevenos J., Halvorsen S. (2018). 2017 ESC Guidelines for the management of acute myocardial infarction in patients presenting with ST-segment elevation. Eur. Heart J..

[44] Panteghini P. (2003). Recommendations on Use of Biochemical Markers in Acute Coronary Syndrome: IFCC Proposals: IFCC Proposals. EJIFCC.

[45] Dekker M., Mosterd A., Van’t Hof A., Hoes A. (2010). Novel biochemical markers in suspected acute coronary syndrome: Systematic review and critical appraisal: Systematic review and critical appraisal. Heart.

[46] Jacob R., Khan M. (2019). Cardiac Biomarkers: What Is and What Can Be. Indian J. Cardiovasc. Dis. Women WINCARS.

[47] Chistiakov D., Orekhov A., Bobryshev Y. (2016). Cardiac-specific miRNA in cardiogenesis, heart function, and cardiac pathology (with focus on myocardial infarction). J. Mol. Cell. Cardiol..

[48] Wang G., Zhu J., Zhang J., Li Q., Li Y., He J., Qin Y., Jing Q. (2010). Circulating microRNA: A novel potential biomarker for early diagnosis of acute myocardial infarction in humans: A novel potential biomarker for early diagnosis of acute myocardial infarction in humans. Eur. Heart J..

[49] Navickas R., Gal D., Laucevičius A., Taparauskaite A., Zdanyte M., Holvoet P. (2016). Identifying circulating microRNAs as biomarkers of cardiovascular disease: A systematic review: A systematic review. Cardiovasc. Res..

[50] Li S., Lee C., Song J., Lu C., Liu J., Cui Y., Liang H., Cao C., Zhang F., Chen H. (2017). Circulating microRNAs as potential biomarkers for coronary plaque rupture. Oncotarget.

[51] Zhong J., He Y., Chen W., Shui X., Chen C., Lei W. (2014). Circulating microRNA-19a as a potential novel biomarker for diagnosis of acute myocardial infarction. Int. J. Mol. Sci..

[52] Zeller T., Tanja T., Ojeda F., Reichlin T., Twerenbold R., Tzikas S., Wild P., Reiter M., Czyz E., Lackner K. (2014). Assessment of microRNAs in patients with unstable angina pectoris. Eur. Heart J..

[53] Gidlöf O., Andersson P., Van Der Pals J., Götberg M., Erlinge D. (2011). Cardiospecific microRNA Plasma Levels Correlate with Troponin and Cardiac Function in Patients with ST Elevation Myocardial Infarction, Are Selectively Dependent on Renal Elimination, and Can Be Detected in Urine Samples. Cardiology.

[54] Oerlemans M., Mosterd A., Dekker M., de Vrey E., van Mil A., Pasterkamp G., Doevendans P., Hoes A., Sluijter J. (2012). Early assessment of acute coronary syndromes in the emergency department: The potential diagnostic value of circulating microRNAs: The potential diagnostic value of circulating microRNAs. EMBO Mol. Med..

[55] Devaux Y., Mueller M., Haaf P., Goretti E., Twerenbold R., Zangrando J., Vausort M., Reichlin T., Wildi K., Moehring B. (2015). Diagnostic and prognostic value of circulating microRNAs in patients with acute chest pain. J. Intern. Med..

[56] Widera C., Gupta S., Lorenzen J., Bang C., Bauersachs J., Bethmann K., Kempf T., Wollert K., Thum T. (2011). Diagnostic and prognostic impact of six circulating microRNAs in acute coronary syndrome. J. Mol. Cell. Cardiol..

[57] Zhang L., Chen X., Su T., Li H., Huang Q., Wu D., Yang C., Han Z. (2015). Circulating miR-499 are novel and sensitive biomarker of acute myocardial infarction. J. Thorac. Dis..

[58] Corsten M., Dennert R., Jochems S., Kuznetsova T., Devaux Y., Hofstra L., Wagner D., Staessen J., Heymans S., Schroen B. (2010). Circulating MicroRNA-208b and MicroRNA-499 reflect myocardial damage in cardiovascular disease. Circ. Cardiovasc. Genet..

[59] Gacon J., Kablak-Ziembicka A., Stepien E., Enguita F., Karch I., Derlaga B., Zmudka K., Przewlocki T. (2016). Decision-making microRNAs (miR-124, -133a/b, -34a and -134) in patients with occluded target vessel in acute coronary syndrome. Kardiol. Pol..

[60] Gao H., Guddeti R., Matsuzawa Y., Liu L., Su L., Guo D., Nie S., Du J., Zhang M. (2015). Plasma Levels of microRNA-145 Are Associated with Severity of Coronary Artery Disease. PLoS ONE.

[61] Matsumoto S., Sakata Y., Nakatani D., Suna S., Mizuno H., Shimizu M., Usami M., Sasaki T., Sato H., Kawahara Y. (2012). A subset of circulating microRNAs are predictive for cardiac death after discharge for acute myocardial infarction. Biochem. Biophys. Res. Commun..

[62] Devaux Y., Vausort M., McCann G., Kelly D., Collignon O., Ng L., Wagner D., Squire I. (2013). A Panel of 4 microRNAs Facilitates the Prediction of Left Ventricular Contractility after Acute Myocardial Infarction. PLoS ONE.

[63] Bye A., Røsjø H., Nauman J., Silva G., Follestad T., Omland T., Wisløff U. (2016). Circulating microRNAs predict future fatal myocardial infarction in healthy individuals—The HUNT study. J. Mol. Cell. Cardiol..

[64] Karakas M., Schulte C., Appelbaum S., Ojeda F., Lackner K., Münzel T., Schnabel R., Blankenberg S., Zeller T. (2017). Circulating microRNAs strongly predict cardiovascular death in patients with coronary artery disease—Results from the large AtheroGene study. Eur. Heart J..

[65] Zampetaki A., Willeit P., Tilling L., Drozdov I., Prokopi M., Renard J., Mayr A., Weger S., Schett G., Shah A. (2012). Prospective Study on Circulating MicroRNAs and Risk of Myocardial Infarction. J. Am. Coll. Cardiol..

[66] Schulte C., Molz S., Appelbaum S., Karakas M., Ojeda F., Lau D., Hartmann T., Lackner K., Westermann D., Schnabel R. (2015). miRNA-197 and miRNA-223 Predict Cardiovascular Death in a Cohort of Patients with Symptomatic Coronary Artery Disease. PLoS ONE.

[67] Tsutsui H., Isobe M., Ito H., Ito H., Okumura K., Ono M., Kitakaze M., Kinugawa K., Kihara Y., Goto Y. (2019). JCS 2017/JHFS 2017 Guideline on Diagnosis and Treatment of Acute and Chronic Heart Failure—Digest Version. Circ. J..

[68] McDonagh T., Metra M., Adamo M., Gardner R., Baumbach A., Böhm M., Burri H., Butler J., Čelutkienė J., Chioncel O. (2021). 2021 ESC Guidelines for the diagnosis and treatment of acute and chronic heart failure. Eur. Heart J..

[69] Wong L., Wang J., Liew O., Richards A., Chen Y. (2016). MicroRNA and Heart Failure. Int. J. Mol. Sci..

[70] Duan Q., Chen C., Yang L., Li N., Gong W., Li S., Wang D. (2015). MicroRNA regulation of unfolded protein response transcription factor XBP1 in the progression of cardiac hypertrophy and heart failure in vivo. J. Transl. Med..

[71] Da Costa Martins P., De Windt L. (2012). MicroRNAs in control of cardiac hypertrophy. Cardiovasc. Res..

[72] Pan Z., Sun X., Ren J., Li X., Gao X., Lu C., Zhang Y., Sun H., Wang Y., Wang H. (2012). miR-1 Exacerbates Cardiac Ischemia-Reperfusion Injury in Mouse Models. PLoS ONE.

[73] Pan W., Zhong Y., Cheng C., Liu B., Wang L., Li A., Xiong L., Liu S. (2013). MiR-30-Regulated Autophagy Mediates Angiotensin II-Induced Myocardial Hypertrophy. PLoS ONE.

[74] Zhu H., Yang Y., Wang Y., Li J., Schiller P., Peng T. (2011). MicroRNA-195 promotes palmitate-induced apoptosis in cardiomyocytes by down-regulating Sirt1. Cardiovasc. Res..

[75] Wang J., Jia Z., Zhang C., Sun M., Wang W., Chen P., Ma K., Zhang Y., Li X., Zhou C. (2014). miR-499 protects cardiomyocytes from H_2_O_2_-induced apoptosis via its effects on *Pdcd4* and *Pacs2*. RNA Biol..

[76] Zhang B., Zhou M., Li C., Zhou J., Li H., Zhu D., Wang Z., Chen A., Zhao Q. (2014). MicroRNA-92a Inhibition Attenuates Hypoxia/Reoxygenation-Induced Myocardiocyte Apoptosis by Targeting Smad7. PLoS ONE.

[77] Sayed D., He M., Hong C., Gao S., Rane S., Yang Z., Abdellatif M. (2010). MicroRNA-21 Is a Downstream Effector of AKT That Mediates Its Antiapoptotic Effects via Suppression of Fas Ligand. J. Biol. Chem..

[78] Tijsen A., Creemers E., Moerland P., Windt L., Wal A., Kok W., Pinto Y. (2010). MiR423-5p as a circulating biomarker for heart failure. Circ. Res..

[79] Goren Y., Kushnir M., Zafrir B., Tabak S., Lewis B., Amir O. (2012). Serum levels of microRNAs in patients with heart failure. Eur. J. Heart Fail..

[80] Tutarel O., Dangwal S., Bretthauer J., Westhoff-Bleck M., Roentgen P., Anker S., Bauersachs J., Thum T. (2013). Circulating miR-423-5p fails as a biomarker for systemic ventricular function in adults after atrial repair for transposition of the great arteries. Int. J. Cardiol..

[81] Scrutinio D., Conserva F., Passantino A., Iacoviello M., Lagioia R., Gesualdo L. (2017). Circulating microRNA-150-5p as a novel biomarker for advanced heart failure: A genome-wide prospective study: A genome-wide prospective study. J. Heart Lung Transplant..

[82] Wong L., Zou R., Zhou L., Lim J., Phua D., Liu C., Chong J., Ng J., Liew O., Chan S. (2019). Combining Circulating MicroRNA and NT-proBNP to Detect and Categorize Heart Failure Subtypes. J. Am. Coll. Cardiol..

[83] Zhang J., Xing Q., Zhou X., Li J., Li Y., Zhang L., Zhou Q., Tang B. (2017). Circulating miRNA-21 is a promising biomarker for heart failure. Mol. Med. Rep..

[84] Masson S., Batkai S., Beermann J., Bär C., Pfanne A., Thum S., Magnoli M., Balconi G., Nicolosi G., Tavazzi L. (2018). Circulating microRNA-132 levels improve risk prediction for heart failure hospitalization in patients with chronic heart failure. Eur. J. Heart Fail..

[85] Seronde M., Vausort M., Gayat E., Goretti E., Ng L., Squire I., Vodovar N., Sadoune M., Samuel J., Thum T. (2015). Circulating microRNAs and outcome in patients with acute heart failure. PLoS ONE.

[86] Van Boven N., Akkerhuis K., Anroedh S., Rizopoulos D., Pinto Y., Battes L., Hillege H., Caliskan K., Germans T., Manintveld O. (2017). Serially measured circulating miR-22-3p is a biomarker for adverse clinical outcome in patients with chronic heart failure: The Bio-SHiFT study: The Bio-SHiFT study. Int. J. Cardiol..

[87] Cakmak H., Coskunpinar E., Ikitimur B., Barman H., Karadag B., Tiryakioglu N., Kahraman K., Vural V. (2015). The prognostic value of circulating microRNAs in heart failure. J. Cardiovasc. Med..

[88] Duong J., Huyen V., Tible M., Gay A., Guillemain R., Aubert O., Varnous S., Iserin F., Rouvier P., François A. (2014). MicroRNAs as non-invasive biomarkers of heart transplant rejection. Eur. Heart J..

[89] Sukma Dewi I., Torngren K., Gidlöf O., Kornhall B., Öhman J. (2013). Altered serum miRNA profiles during acute rejection after heart transplantation: Potential for non-invasive allograft surveillance: Potential for non-invasive allograft surveillance. J. Heart Lung Transplant..

[90] Peterlin A., Počivavšek K., Petrovič D., Peterlin B. (2020). The Role of microRNAs in Heart Failure: A Systematic Review: A Systematic Review. Front. Cardiovasc. Med..

